# Supplementary material for: Rapid fabrication of complex nanostructures using room-temperature ultrasonic nanoimprinting
Source: Nat Commun. 2021 May 25;12:3146. doi: 10.1038/s41467-021-23427-y (PMC8149427; doi:10.1038/s41467-021-23427-y)
Supplement: Supplementary file 1 — Supplementary Information [file 41467_2021_23427_MOESM1_ESM.pdf]

SUPPLEMENTARY INFORMATION

Rapid Fabrication of Complex Nanostructures using Room-Temperature Ultrasonic Nanoimprinting

Junyu Ge[1]†, Bin Ding[2,3]†, Shuai Hou[4]†, Manlin Luo[5], Donguk Nam[5], Hongwei Duan[4]*, Huajian Gao[1,3]*, Yee Cheong Lam[1]*, Hong Li[1,5,6]*

[1]School of Mechanical and Aerospace Engineering, Nanyang Technological University, Singapore 639798, Singapore

[2]Institute of Solid Mechanics, Beihang University, Beijing 100191, P. R. China

[3]Institute of High Performance Computing, A*STAR, Singapore 138632, Singapore

[4]School of Chemical and Biomedical Engineering, Nanyang Technological University, Singapore 637457, Singapore

[5]School of Electrical and Electronic Engineering, Nanyang Technological University, Singapore 639798, Singapore

[6]CINTRA CNRS/NTU/THALES, UMI 3288, Research Techno Plaza, 637553, Singapore

† equal contribution

*corresponding authors. hduan@ntu.edu.sg; Huajian.gao@ntu.edu.sg; myclam@ntu.edu.sg; ehongli@ntu.edu.sg

Supplementary Figures

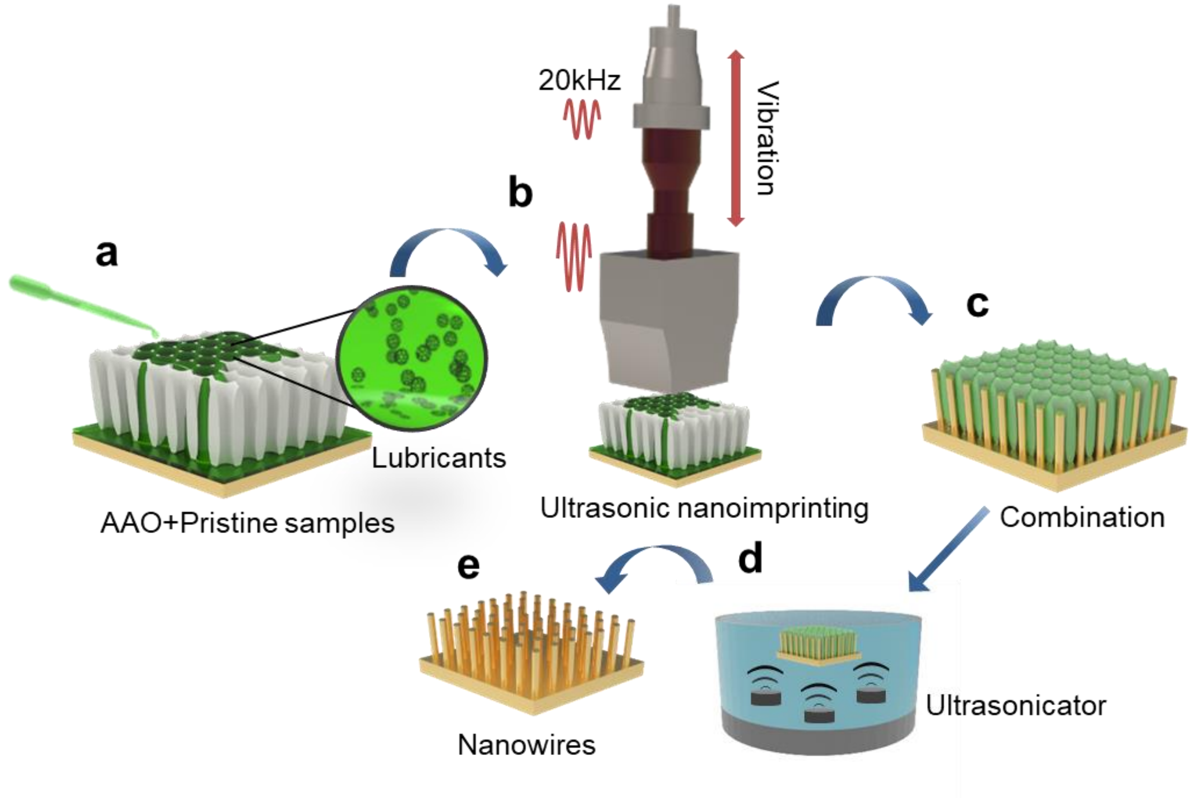

Supplementary Fig. 1 | Working principle of ultrasonic nanoimprinting with demolding process. a, Lubricants dropped on AAO mold. **b,** Ultrasonic nanoimprinting process. **c,** Stock of mold and metal foil. **d,** Ultrasonic assisted AAO mold and nanowires separation process. **e,** Nanowires.

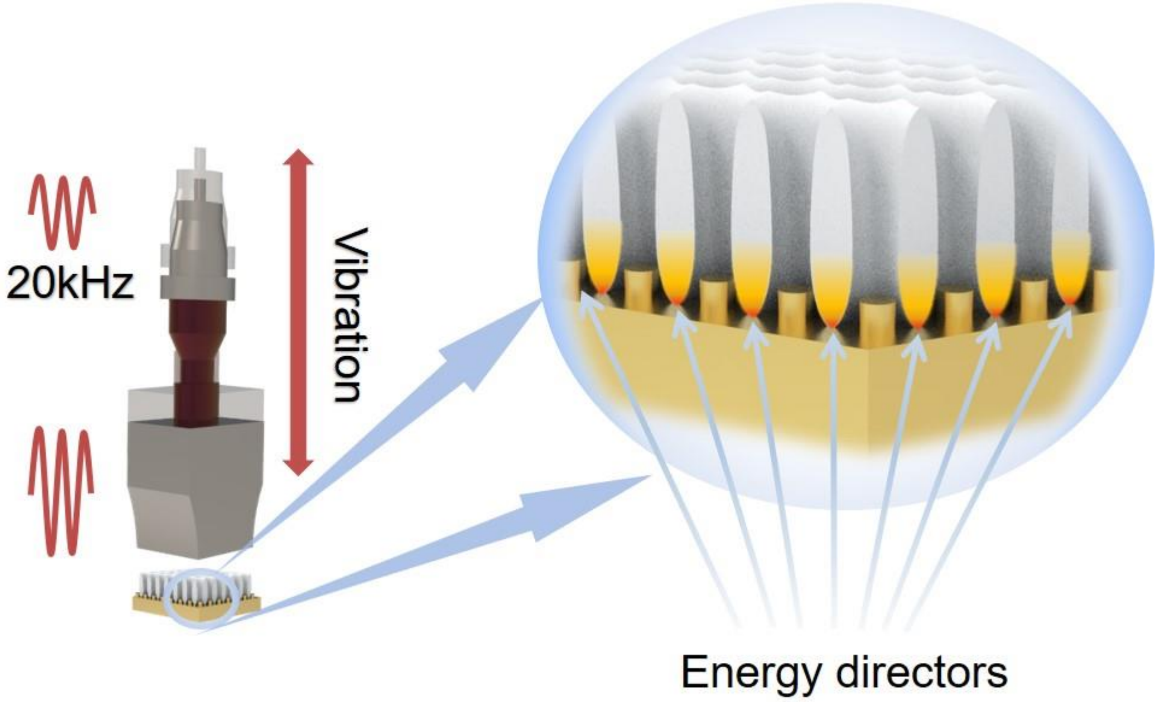

Supplementary Fig. 2 | Working principle of ultrasonic nanoimprinting. The nanoscale walls in AAO mold served as the ‘energy directors’ to deform metal film during the imprinting process.

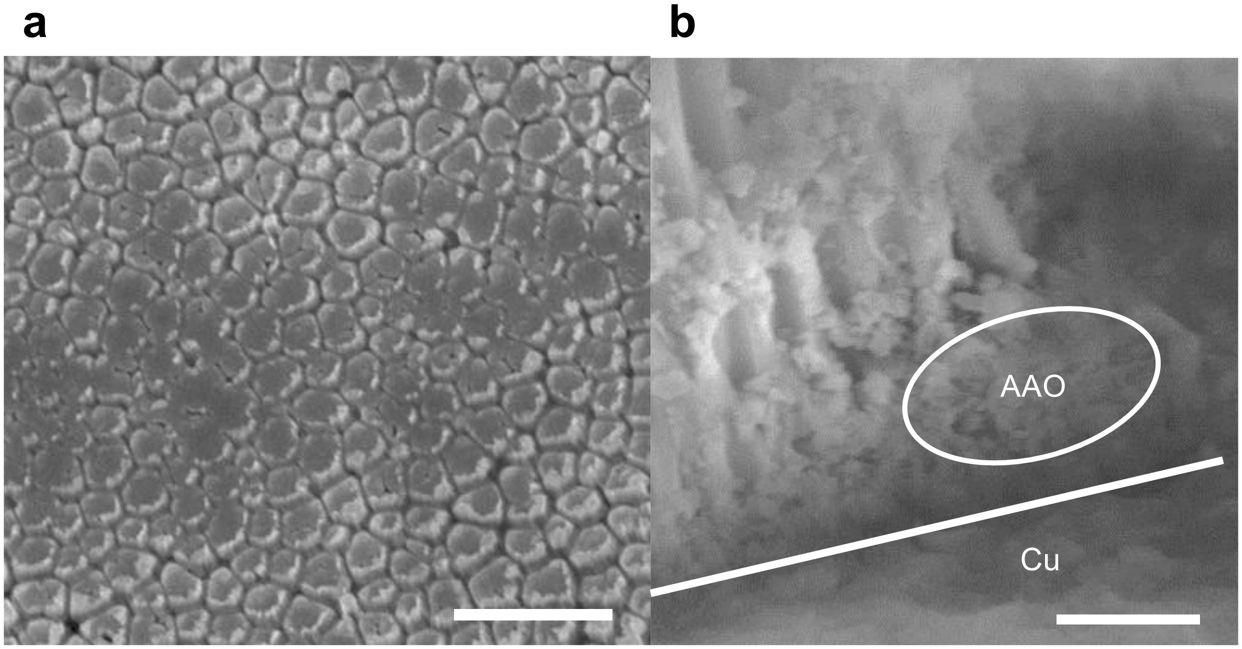

Supplementary Fig. 3 | Direct nanoimprinting on Cu foil. a, SEM image of Cu foil with loading force of 88,000 N. **b,** SEM image of Cu foil and AAO mold when the loading force is 250,000 N, AAO mold was broken, the broken AAO mold damaged Cu foil surface, therefore, no nanowires can be fabricated even under this loading force. Scale bars, 1 μm (**a**,**b**)

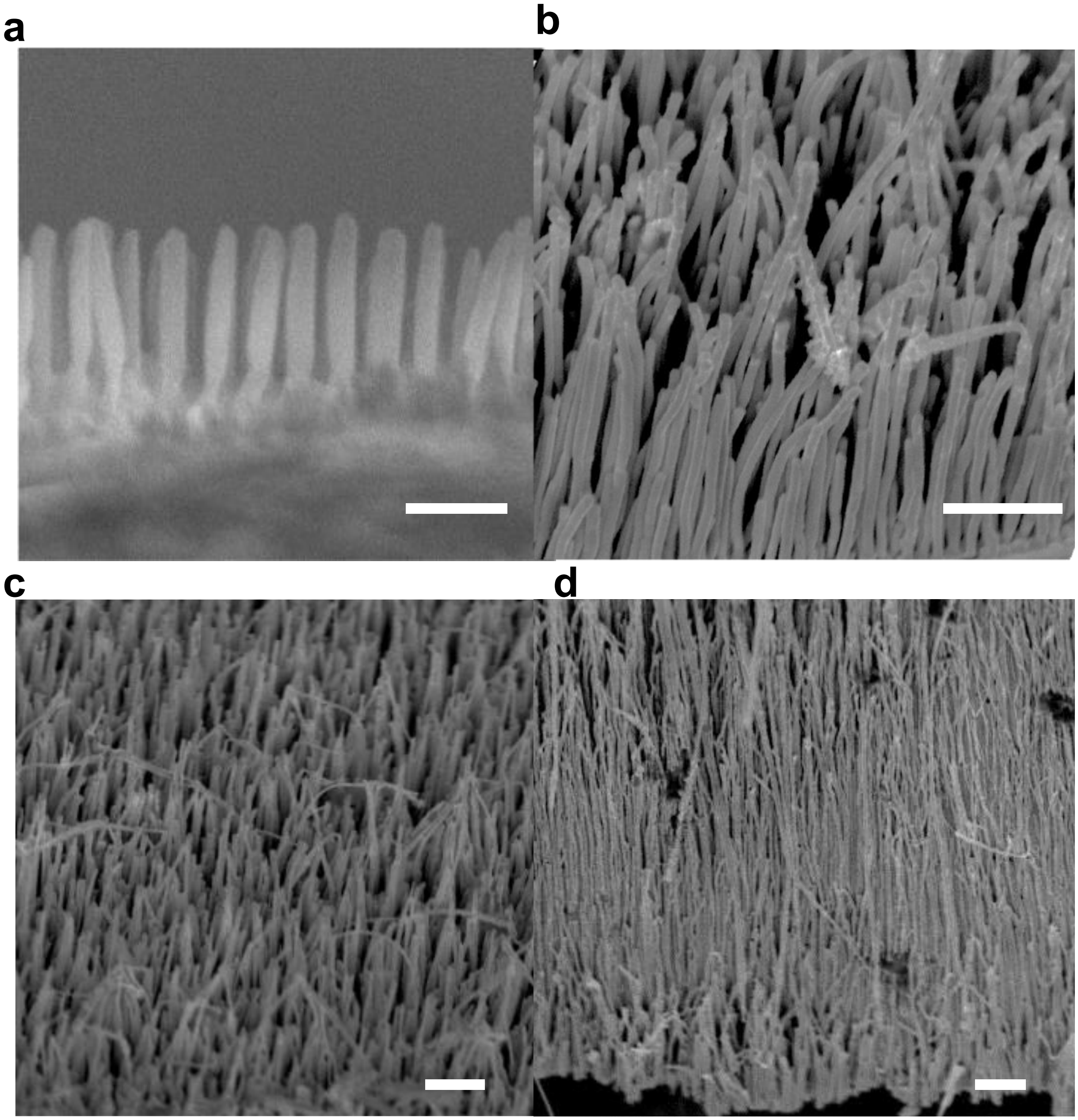

Supplementary Fig. 4 | SEM images of different length Ag nanowires. The lengths of (**a-d**) are 200 nm, 2 µm, 5 µm, and > 10 µm, respectively. Scale bars, 100 nm (**a**) and 1 µm (**b-d**).

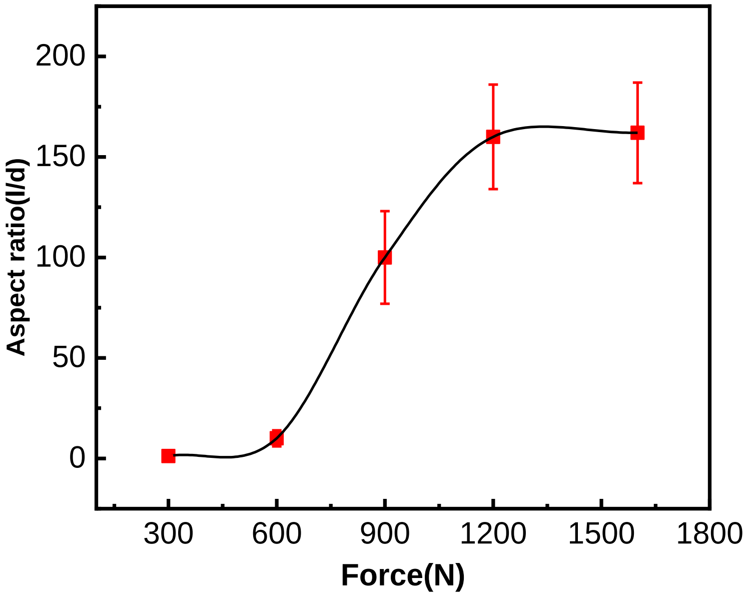

Supplementary Fig. 5 | Processing force dependent aspect ratio of Ag nanowires. The length of Ag nanowires versus force, where four silver foils with thickness of 100 μm were formed into 50-μm-thick AAO mold with pore diameter of 300 nm, under a certain amplitude 20%. Error bars represent standard deviation from 10 data points.

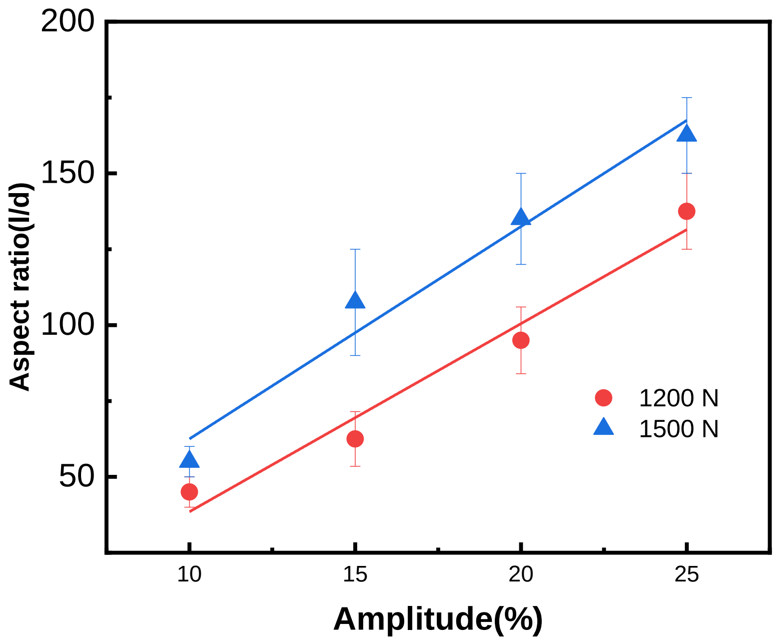

Supplementary Fig. 6 | Vibration amplitude dependent aspect ratio of Ag nanowires. The length of Ag nanowires versus amplitude, where four silver foils with thickness of 100 μm were formed into 50-μm-AAO template with pore diameter of 300 nm, under applied forces of 1200 N and 1500 N, respectively. The process time was 30 sec. Error bars represent standard deviation from 10 data points.

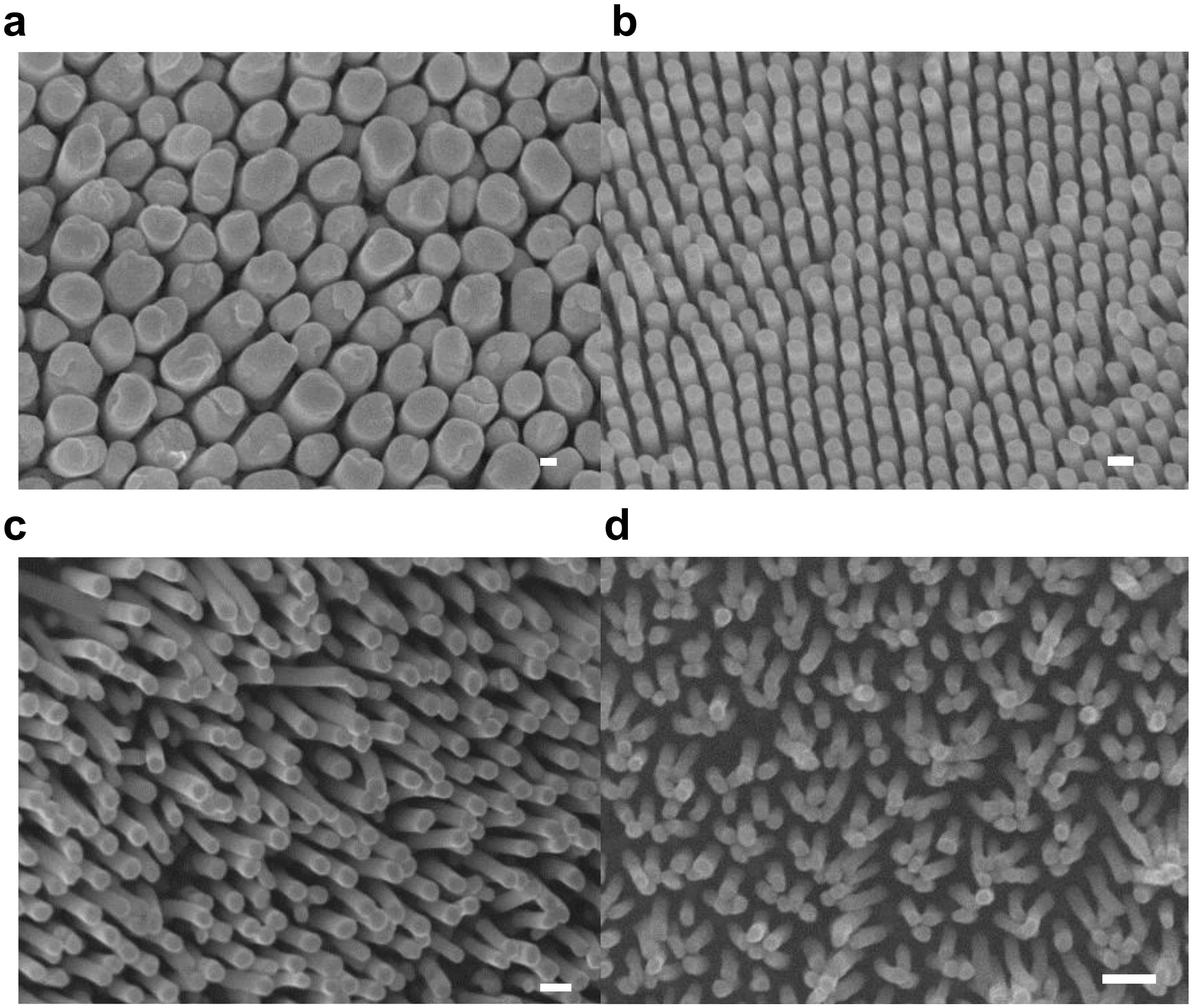

Supplementary Fig. 7 | SEM images of different diameter Au nanowires. The diameter of (**a-d**) are 300 nm, 80 nm, 50 nm, 20 nm, respectively. Scale bars, 100 nm (**a-d**).

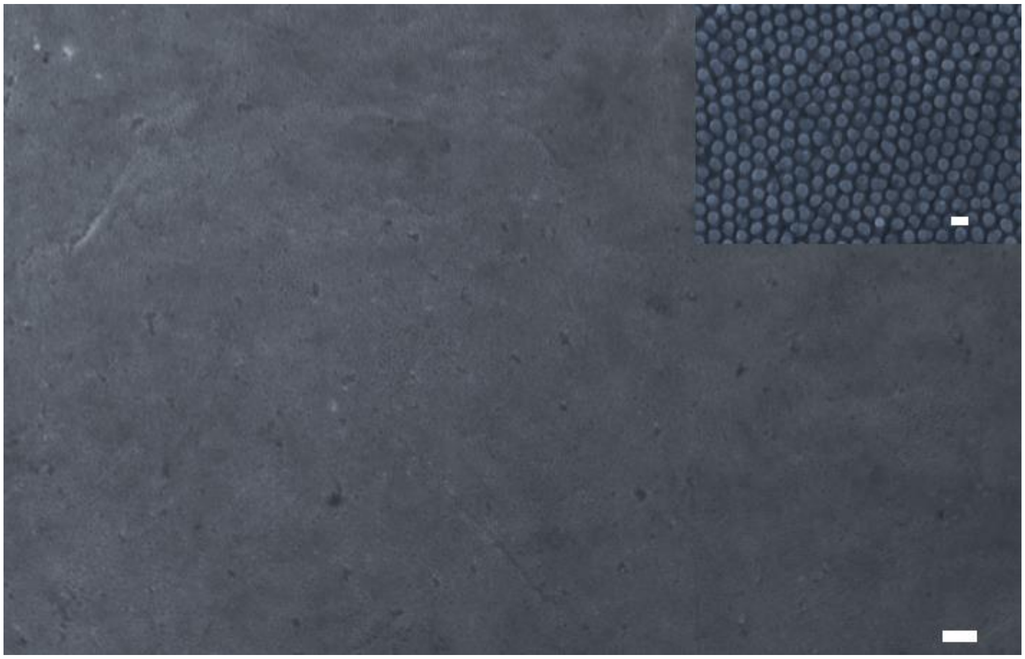

Supplementary Fig. 8 | SEM image of fabricated Au nanowires on silicon wafer. Before ultrasonic nanoimprinting process, 200 nm-thick Au layer was deposited on silicon wafer. Scale bar, 1 μm and 100 nm (inset).

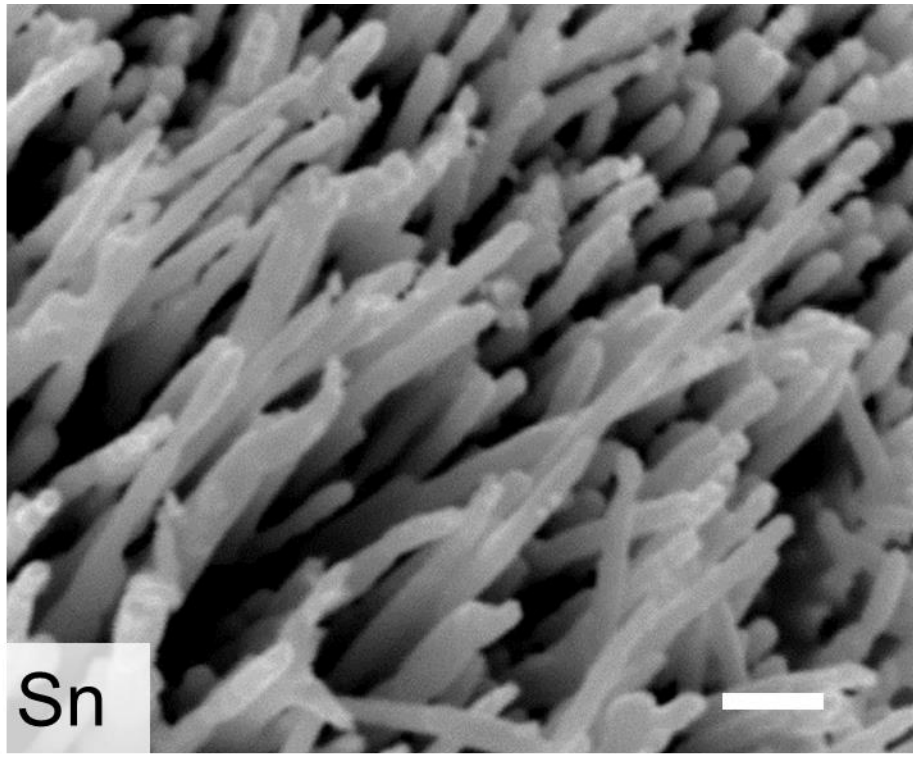

Supplementary Fig. 9 | SEM image of fabricated Sn nanowires. Scale bar, 1 μm.

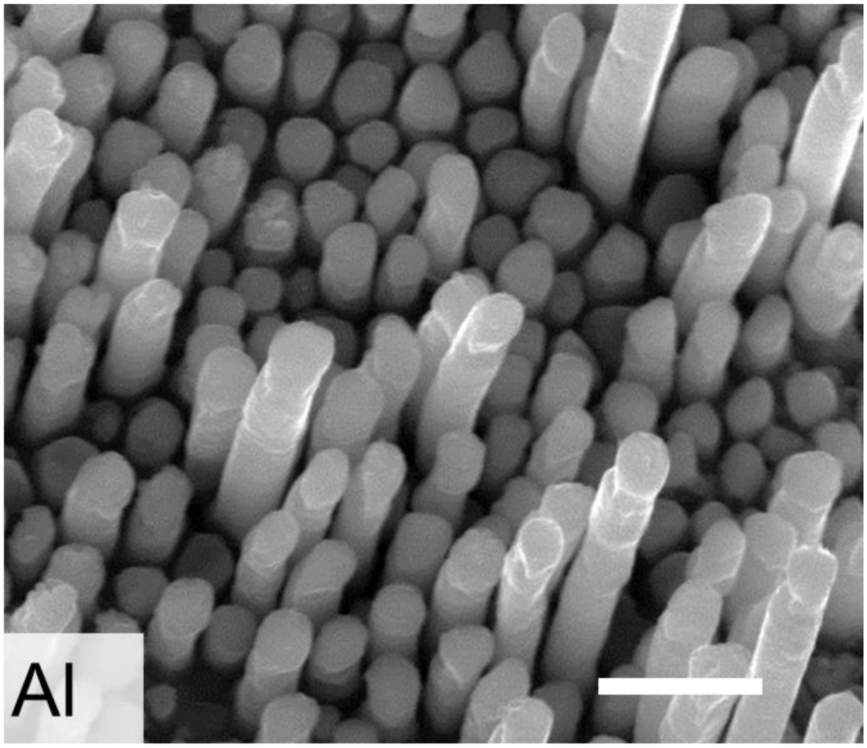

Supplementary Fig. 10 | SEM image of fabricated Al nanowires. Scale bar, 1 μm.

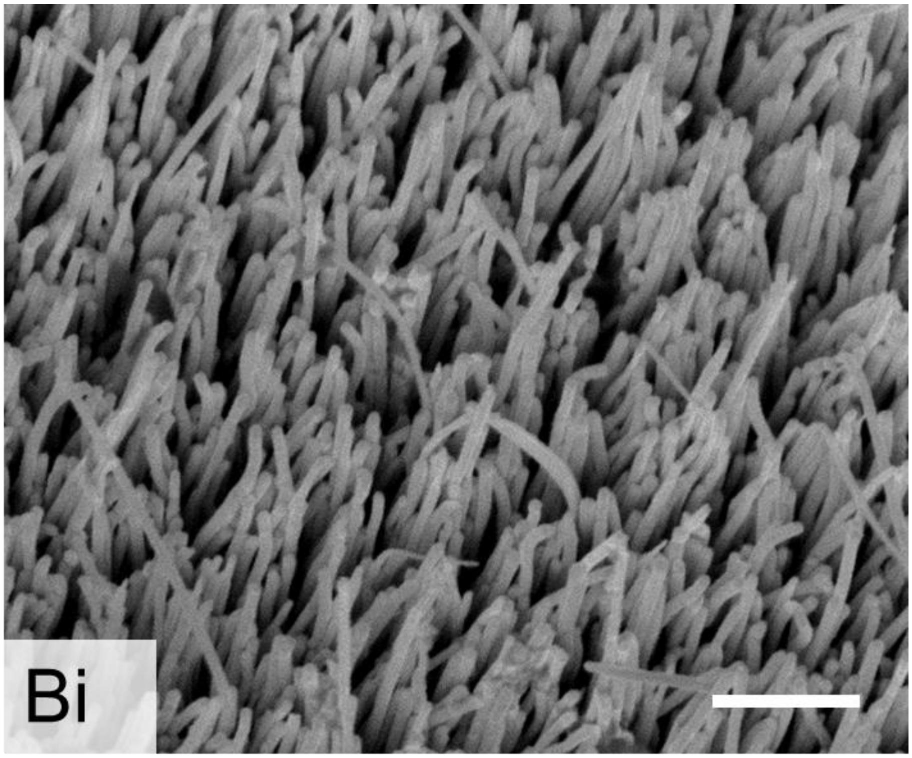

Supplementary Fig. 11 | SEM image of Bi nanowires. Scale bar, 1 μm.

a

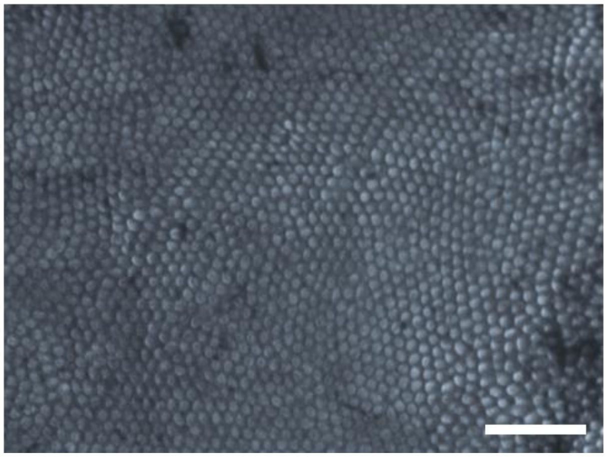

b

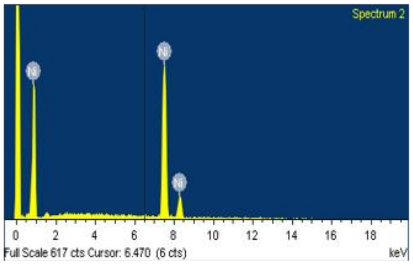

c

Element	Weight (%)	Atomic (%)
Ni K	100.00	100.00
Totals	100.00	100.00

Supplementary Fig. 12 | SEM image and EDS spectra of fabricated Ni nanowires. a, morphology of Ni nanowires. **b,** EDS spectra of the full area in (**a**). **c,** the summary table the element distribution. Scale bar, 2 μm (**a**).

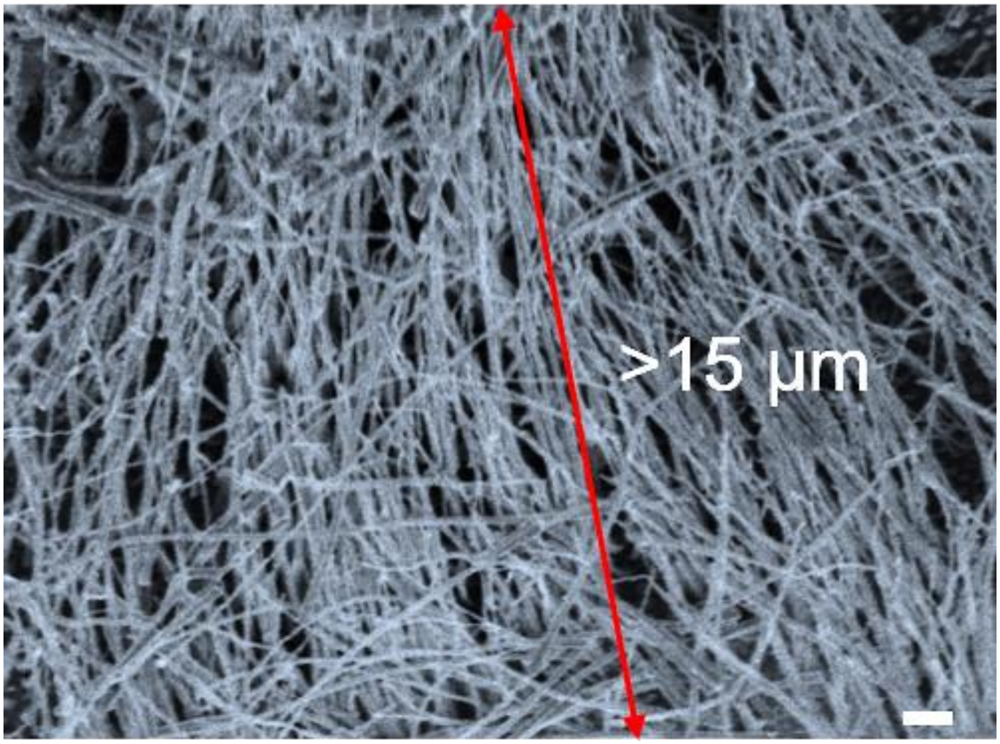

Supplementary Fig. 13 | SEM image of ultrasonic imprinted long Cu nanowires. Scale bar, 1 μm. Long Cu nanowires (> 15 μm) can be obtained under 1,200 N in ultrasonic nanoimprinting while; in apparent contrast, only a dent was made on Cu foil under 88,000 N in direct loading nanoimprinting (see Supplementary Fig. 3).

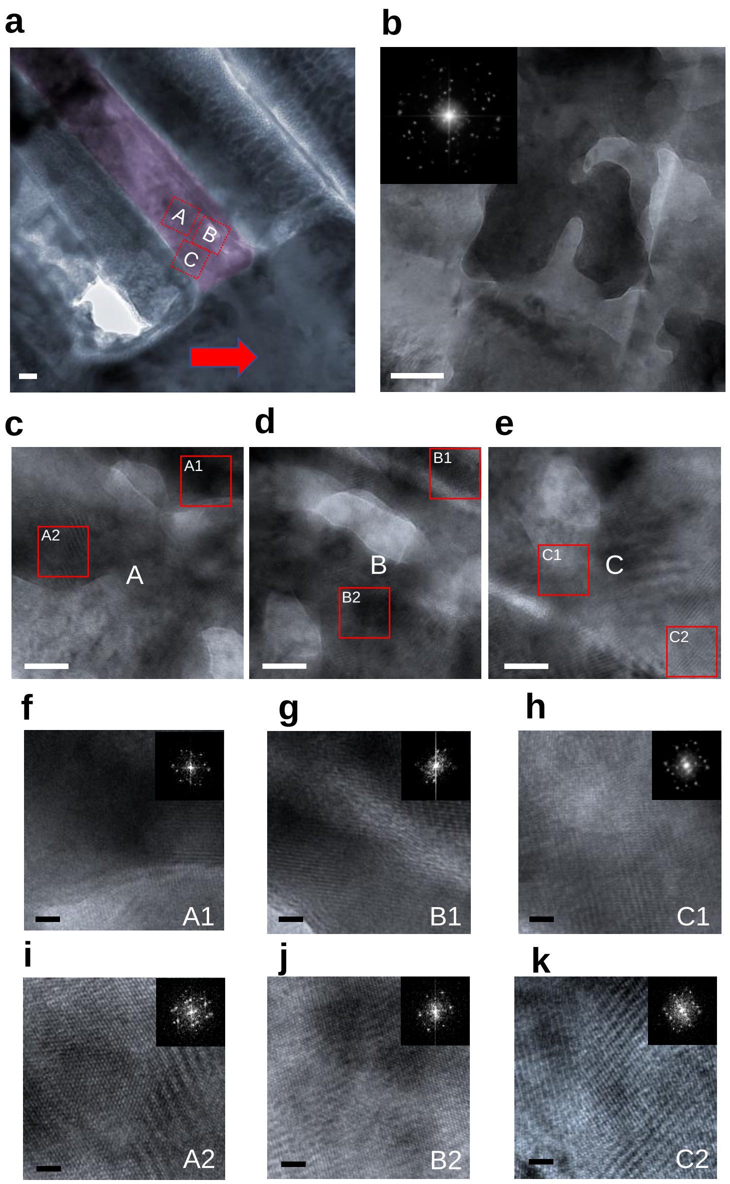

Supplementary Fig. 14 | TEM and HRTEM images of polycrystal Ag nanowire. a, Ag nanowires rooted on Ag foil fabricated by focused ion beam (FIB). **b,** Topography of the bulk Ag foil, the red arrow in (**a**) points to the area of (**b**). The inserted fast Fourier transformation (FFT) image shows the polycrystal nature of Ag foil. **c-e,** TEM images at the regions denoted by A, B, and C in (**a**). **f-k,** HRTEM images of selected area in (**c-e**), the spots of the inserted FFT images are different from each other, indicated the polycrystal nature of the Ag nanowire, which proved the fabricated nanowires retain the crystallinity of bulk materials. Scale bars, 20 nm (**a**,**b**), 10 nm (**c**-**e**), and 2 nm (**f**-**k**).

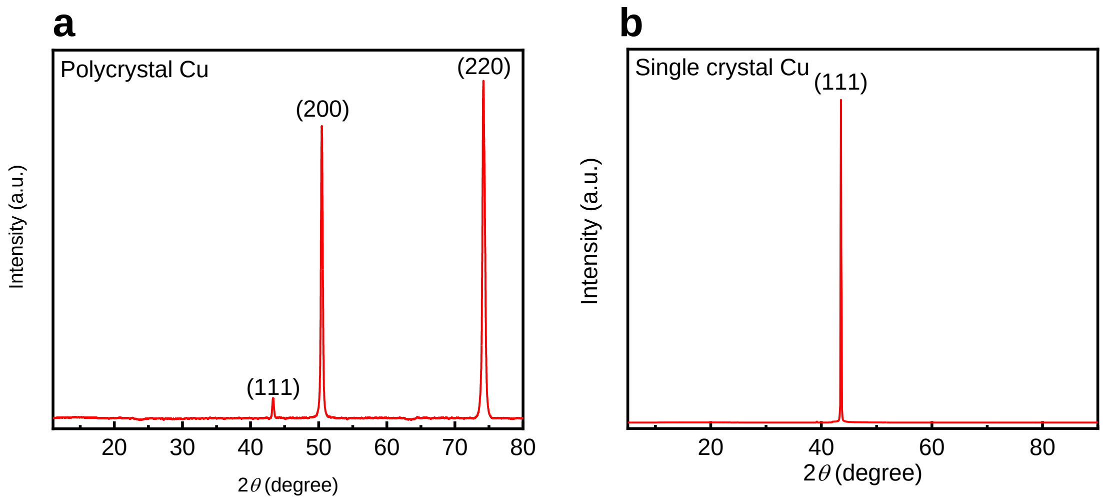

Supplementary Fig. 15 | Identification of pristine crystal Cu foils. X-ray diffraction spectra of (**a**) polycrystal Cu foil and (**b**) single crystal Cu foil.

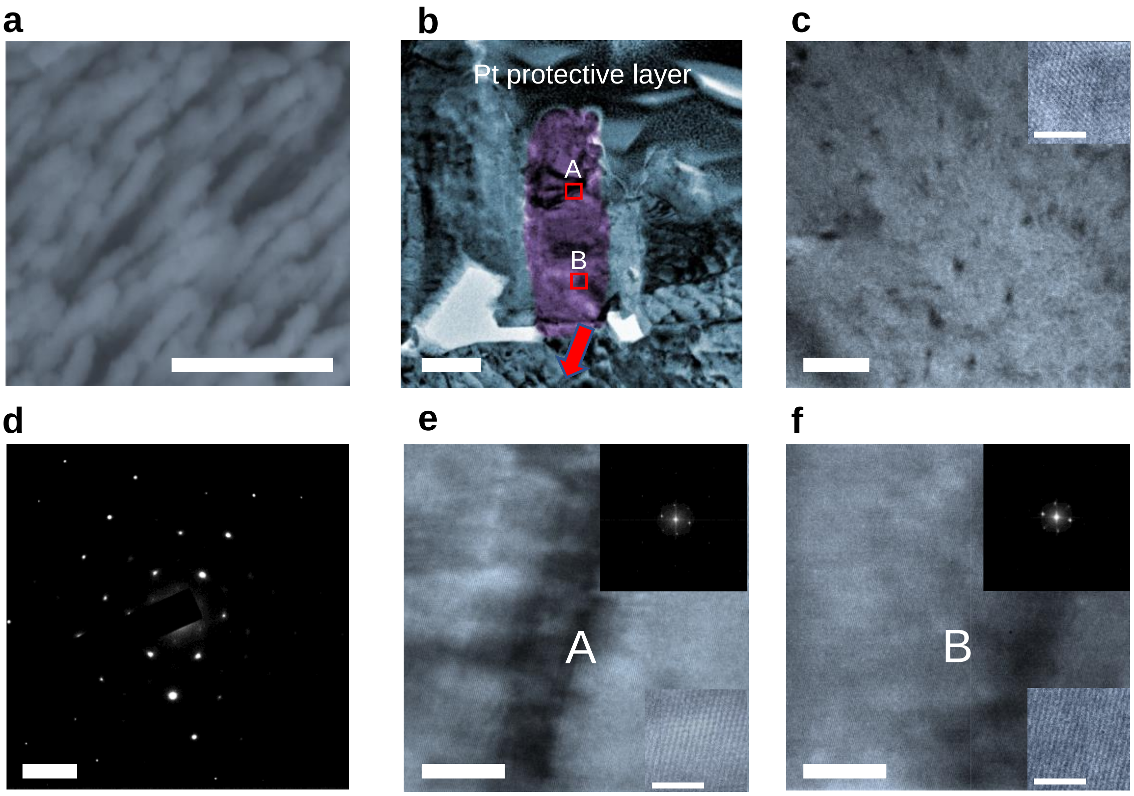

Supplementary Fig. 16 | TEM and HRTEM images of single crystal Cu nanowire. a, SEM image of single crystal Cu nanowires, **b,** TEM image of Cu nanowires rooted on Cu foil fabricated by FIB. The cover layer on the nanowires is Pt layer, which is used to protect the nanowires during FIB cutting process. **c,** Topography of the bulk Cu foil, the red arrow in (**b**) points to the area of (**c**). The inserted clear HRTEM image shows ordered atoms of Cu foil. **d,** Diffraction pattern of the Cu foil in b, which shows a typical FCC single crystal structure. **e,f,** TEM images at the regions denoted by A and B in (**b**). The inserted FFT images of (**e**) and (**f**) indicated the single crystal nature of the Cu nanowire as well. The inserted HRTEM shows the ordered atoms of Cu nanowires. Scale bars, 1 μm (**a**), 100 nm (**b**), and 10 nm (**c, e, and f**), 2 nm (inserted HRTEM in **c, e, f**) and 10 nm^{-1} (**d**).

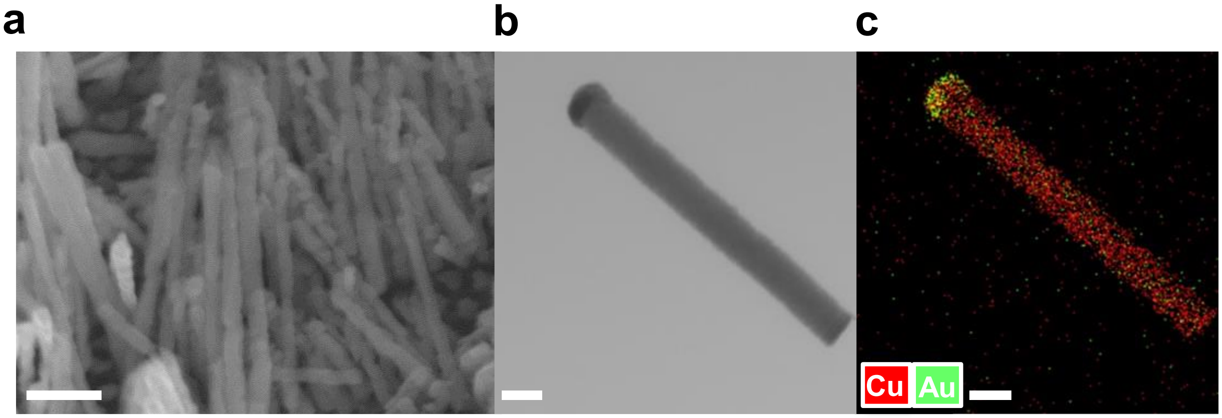

Supplementary Fig. 17 | SEM and TEM images of Au-Cu heterojunctions. a, SEM images of Au-Cu heterojunctions. **(b)** STEM and **(c)** EDS images of a single Au-Cu heterojunction. Scale bars, 1 μm (**a**) and 100 nm (**b,c**).

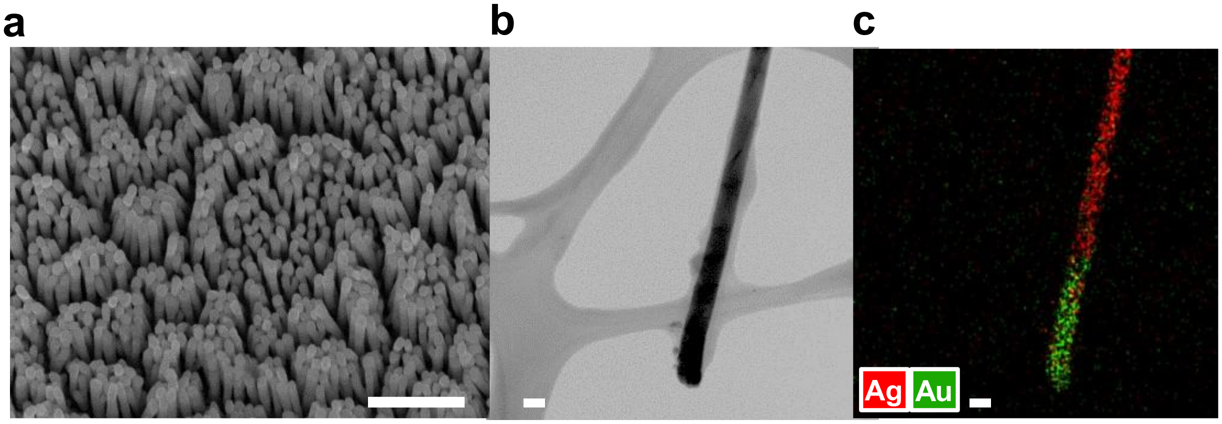

Supplementary Fig. 18 | SEM and TEM images of Au-Ag heterojunctions. a, SEM images of Au-Ag heterojunctions. STEM (**b**) and EDS (**c**) images of a single Au-Ag heterojunction. Scale bars, 1 μm (**a**) and 100 nm (**b,c**).

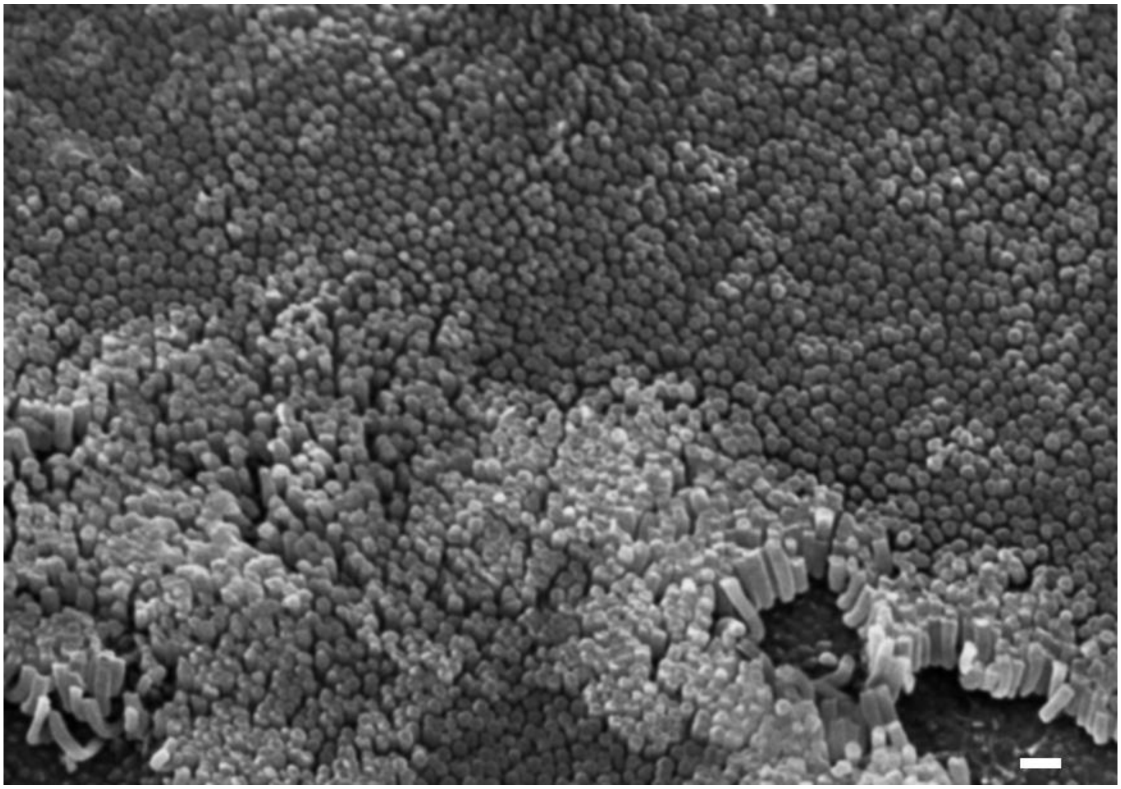

Supplementary Fig. 19 | SEM image of Au-plastic (polycarbonate) heterojunctions. Scale bar, 1 μm.

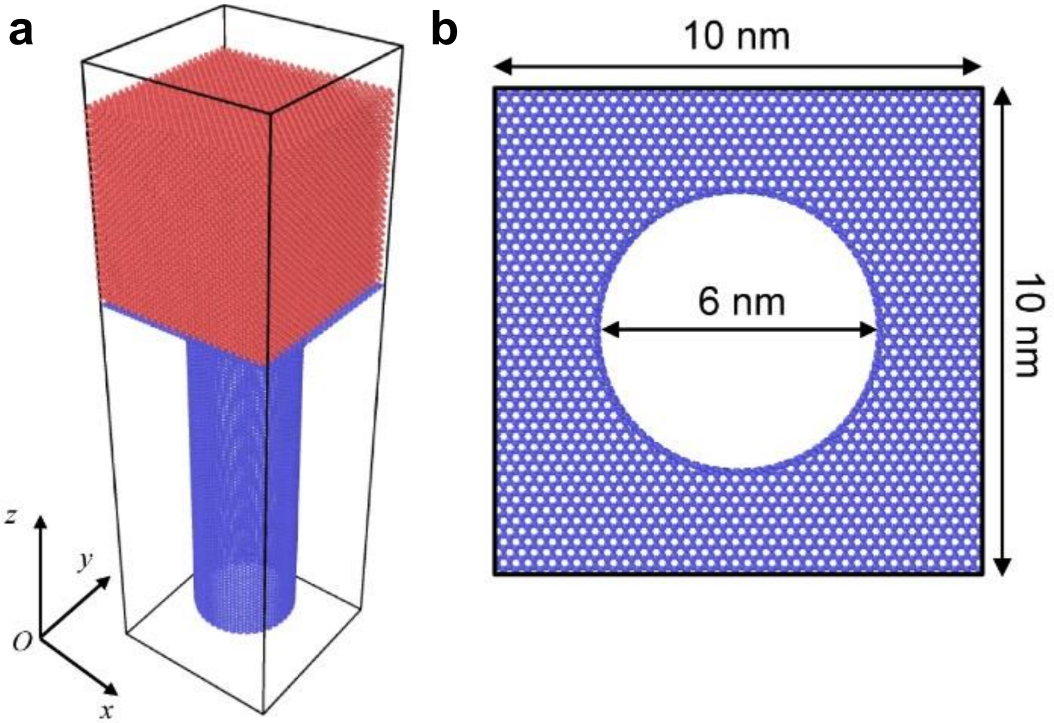

Supplementary Fig. 20 | Model setup of atomistic simulations. a, Atomic configurations of the metal substrate (red atoms) placed on top of the mold (blue atoms). The mold was mimicked by a single layer graphene with a center hole connected to a carbon nanotube (CNT). The mold was kept rigid throughout the imprinting process. The system was periodic in *x*- and *y*-directions. **b,** Top view of the mold.

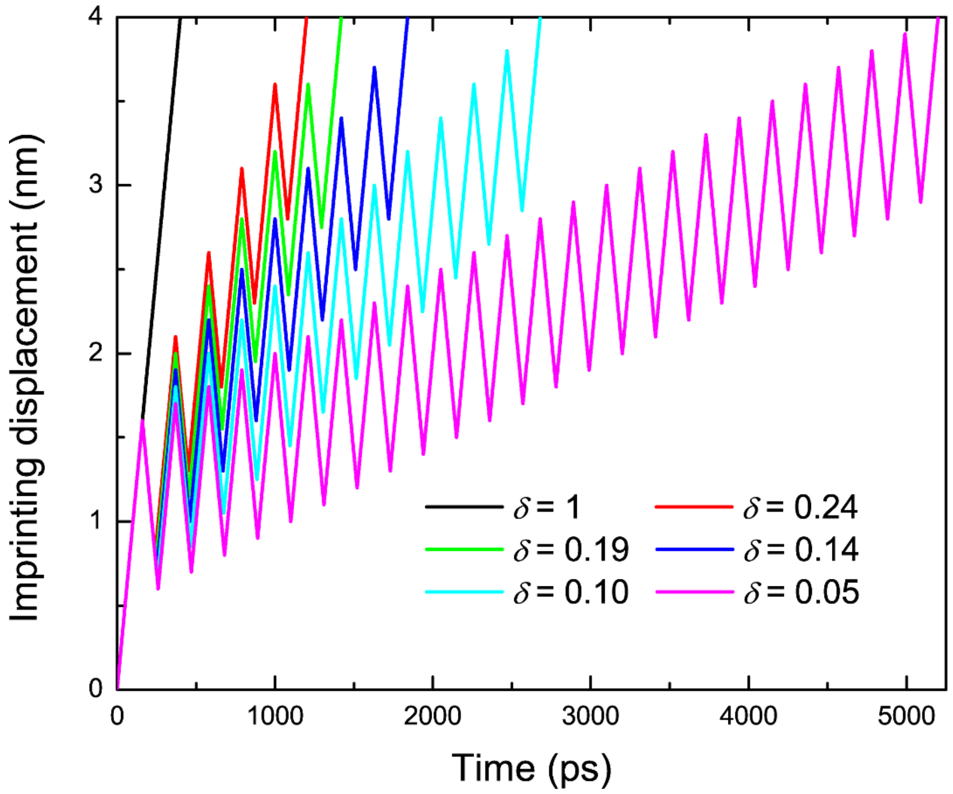

Supplementary Fig. 21 | Selected loading patterns of atomistic simulations. The black line represents the direct loading ($\delta = 1$) without the retreating half-cycles. Five colored curves represent cyclic loadings ($0 < \delta < 1$) with the same frequency but different net imprinting displacements per cycle. The imprinting displacement in loading half-cycle is set to be positive, while in retreating half-cycle is negative.

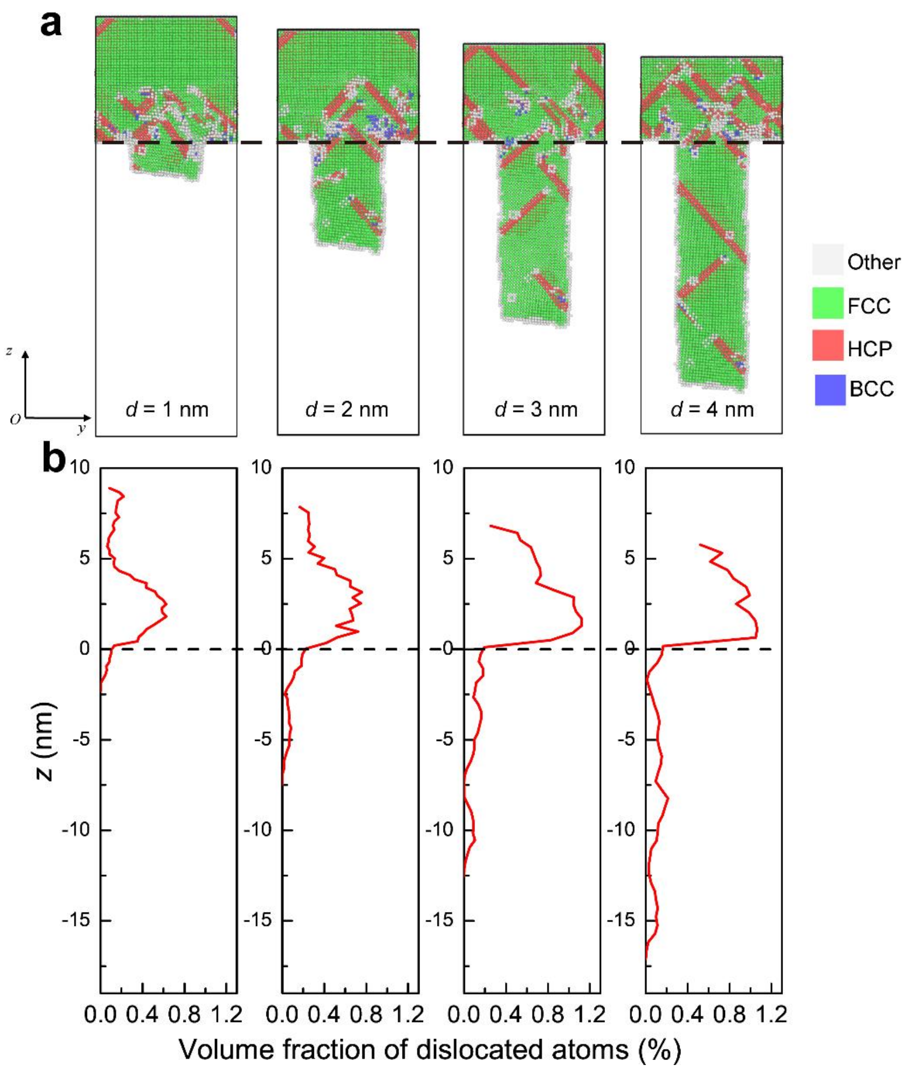

Supplementary Fig. 22 | Snapshots of the deformed silver substrate under direct loading ($\delta = 1$). a Sectional views and **b** volume fraction of dislocated atoms (HCP and BCC type) along the length (*z*-axis) of the imprinted nanowire at moments of the loading displacement *d* = 1, 2, 3, 4 nm. It can be observed that as the imprinting displacement increases, dislocations nucleate, glide, accumulate, pile-up and interact in a continuous manner.

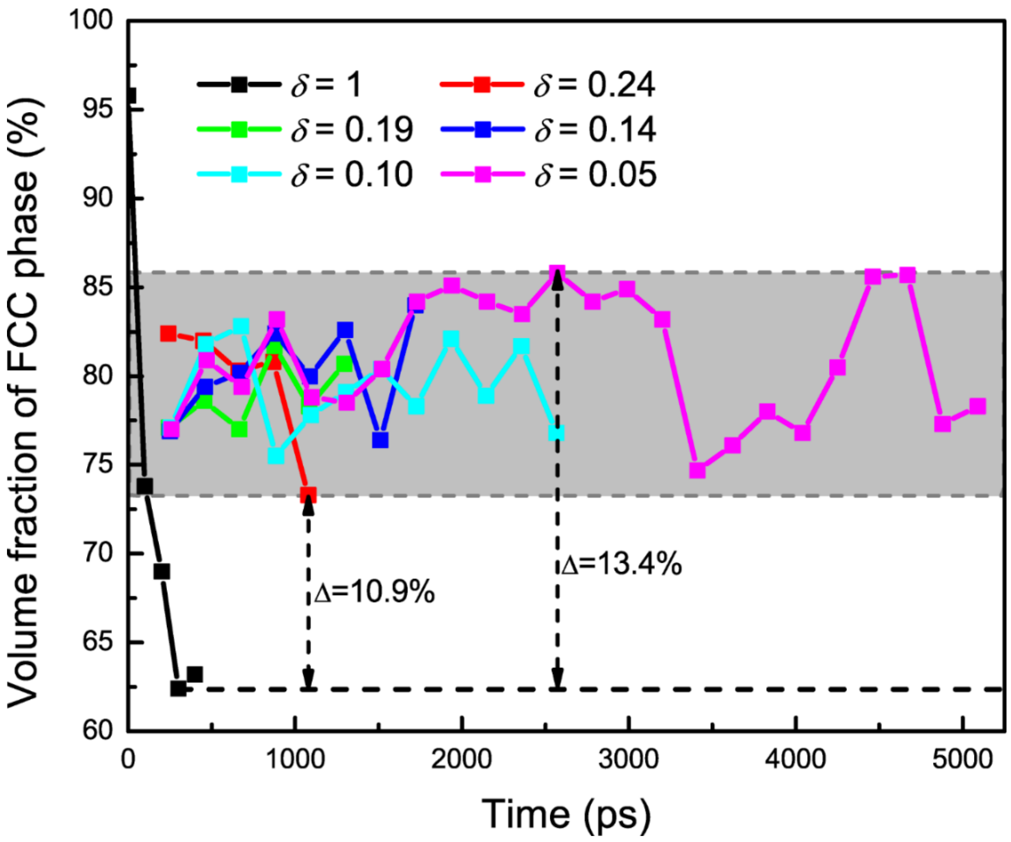

Supplementary Fig. 23 | Volume fraction of FCC phase of the deformed silver substrate under direct and cyclic loadings. Under direct loading ($\delta = 1$), volume fractions of FCC phase are monitored at moments corresponding to imprinting displacement d = 0, 1, 2, 3, 4 nm. Under cyclic loadings ($0 < \delta < 1$), volume fractions of FCC phase are recorded at the end of each loading -retreating cycle. Overall, the volume fraction of FCC phase keeps decreasing under direct loading while remaining within a certain range (10.9%-13.4% higher) under cyclic loadings.

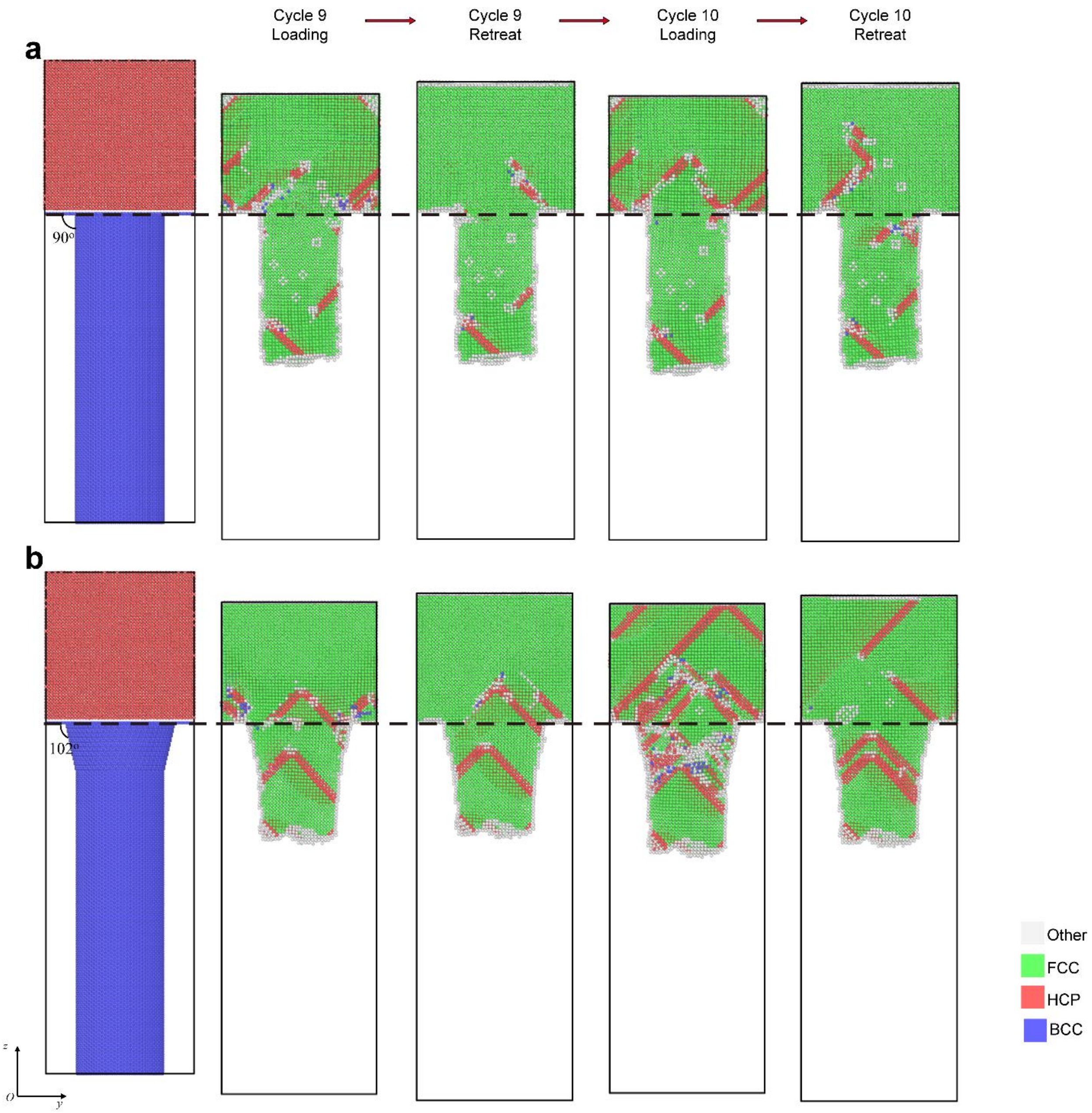

Supplementary Fig. 24 | Comparison of different mold shapes. Sectional views of the deformed Ag substrate confined by the mold with the corner of **(a)** right angle and **(b)** obtuse angle at imprinting cycles 9 and 10 under δ = 0.05. Similar dislocation generation and recovery mechanism is observed in both cases.

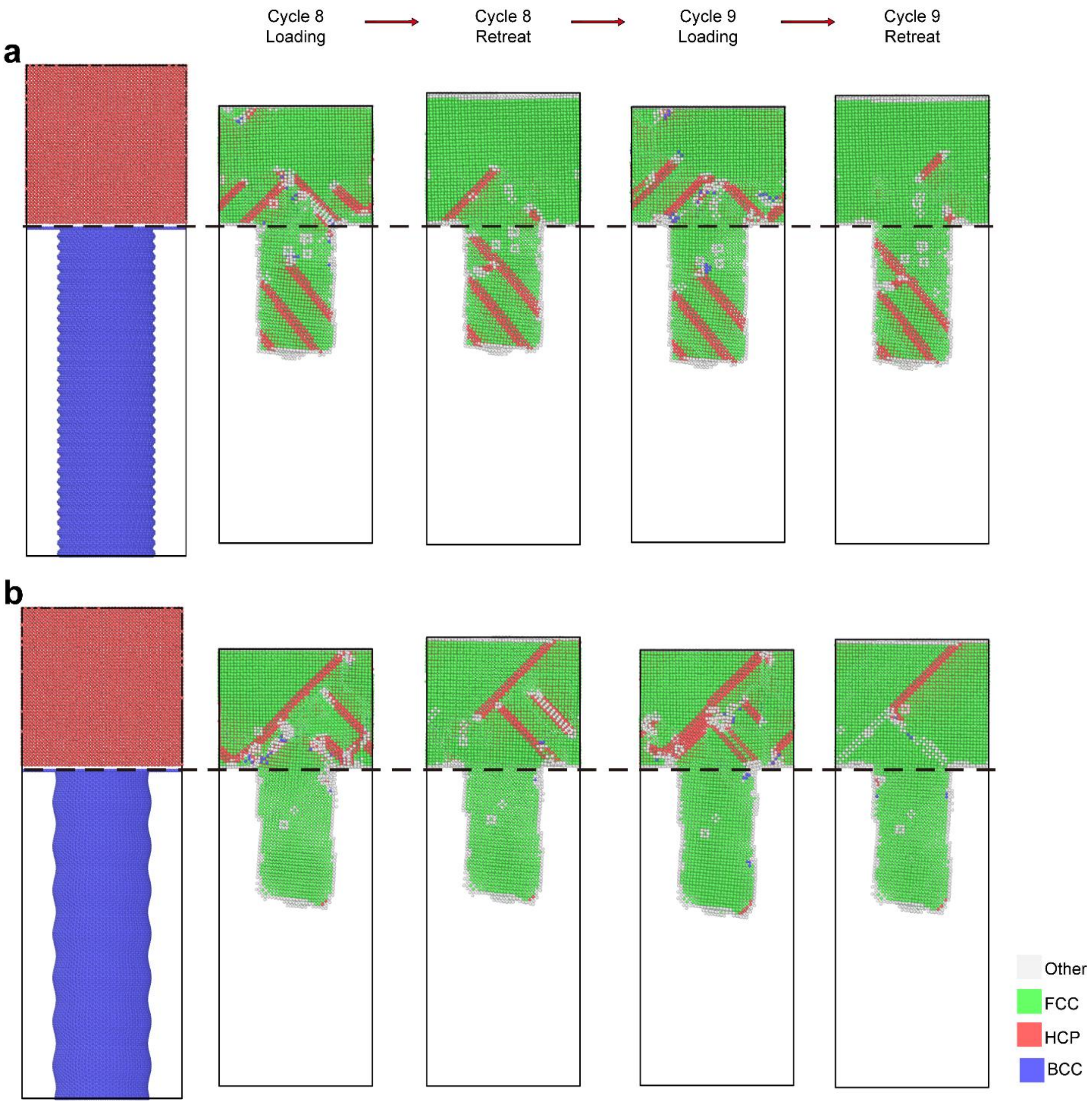

Supplementary Fig. 25 | Comparison of different mold morphologies. Sectional views of the deformed Ag substrate confined by a mold with surface wavelength of (**a**) 0.1 nm and (**b**) 1 nm at imprinting cycles 8 and 9 under δ = 0.05. Similar dislocation generation and recovery mechanism is observed in both cases.

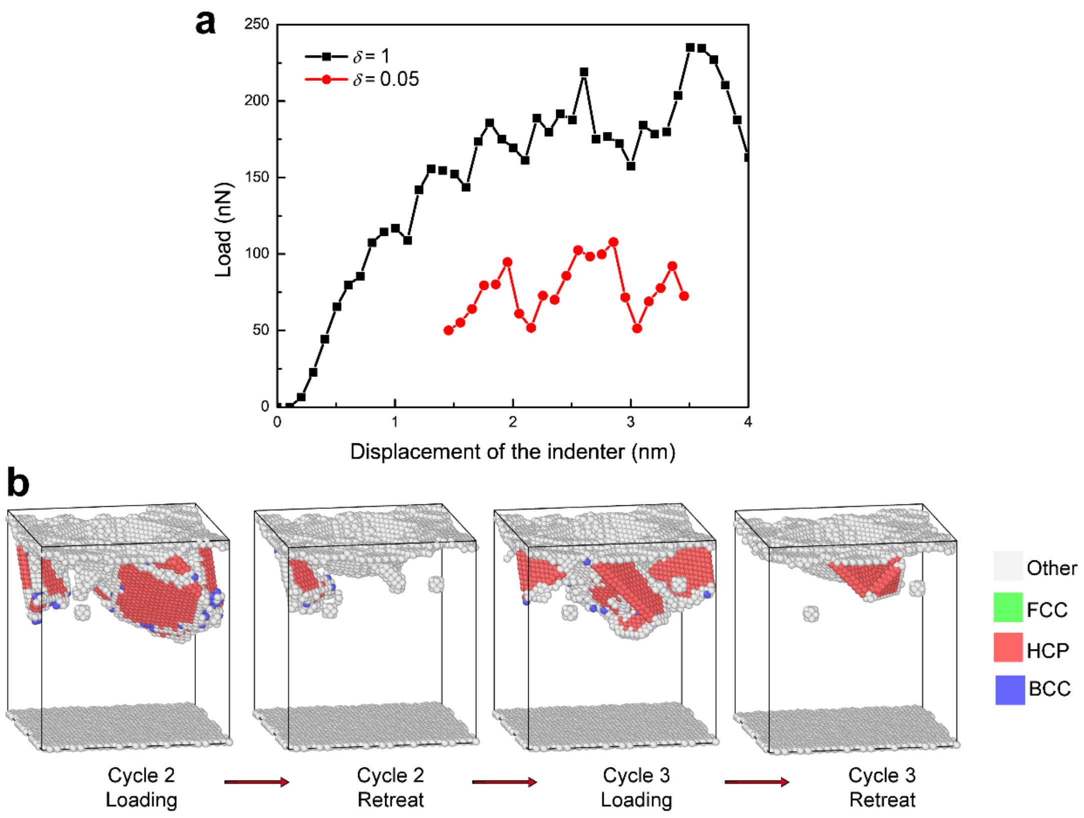

Supplementary Fig. 26 | Atomistic simulations of the nanoindentation at T = 300 K. a, Load-displacement curves under direct loading ($\delta = 1$) and cyclic loading ($\delta = 0.05$); **b,** Snapshots of the deformed Ag substrate at indentation cycles 2 and 3 under $\delta = 0.05$. Atoms with FCC symmetry are removed to clearly show the dislocations. Note that similar dislocation generation and recovery mechanism to that during nanoimprinting also occurred in standard indentation tests under cyclic loading at T = 300 K.

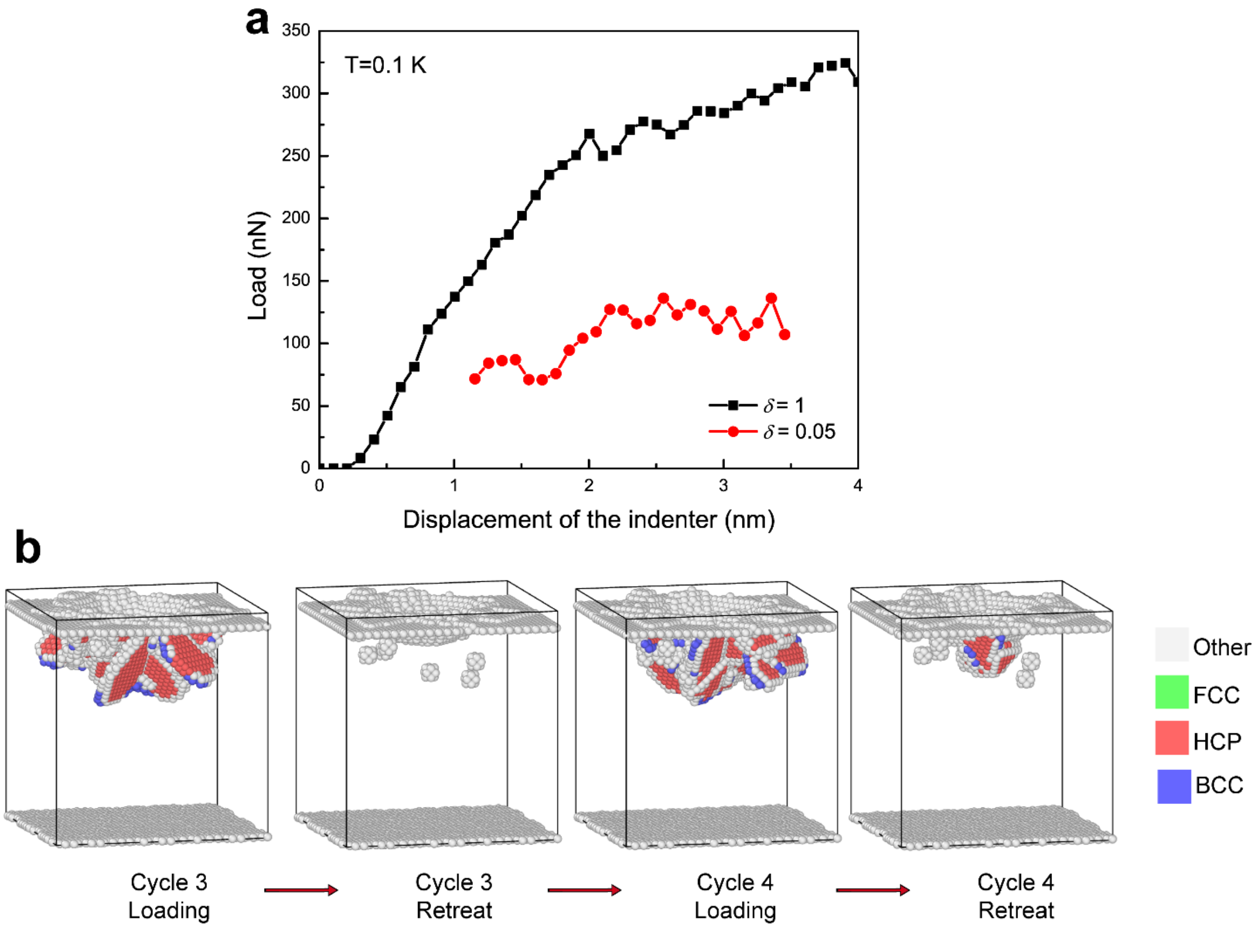

Supplementary Fig. 27 | Atomistic simulations of the nanoindentation at T = 0.1 K. a, Load-displacement curves under direct loading ($\delta = 1$) and cyclic loading ($\delta = 0.05$); **b,** Snapshots of the deformed Ag substrate at indentation cycles 3 and 4 under $\delta = 0.05$. Atoms with FCC symmetry are removed to clearly show the dislocations. Similar dislocation generation and recovery mechanism occurred under cyclic loading at T = 0.1 K.

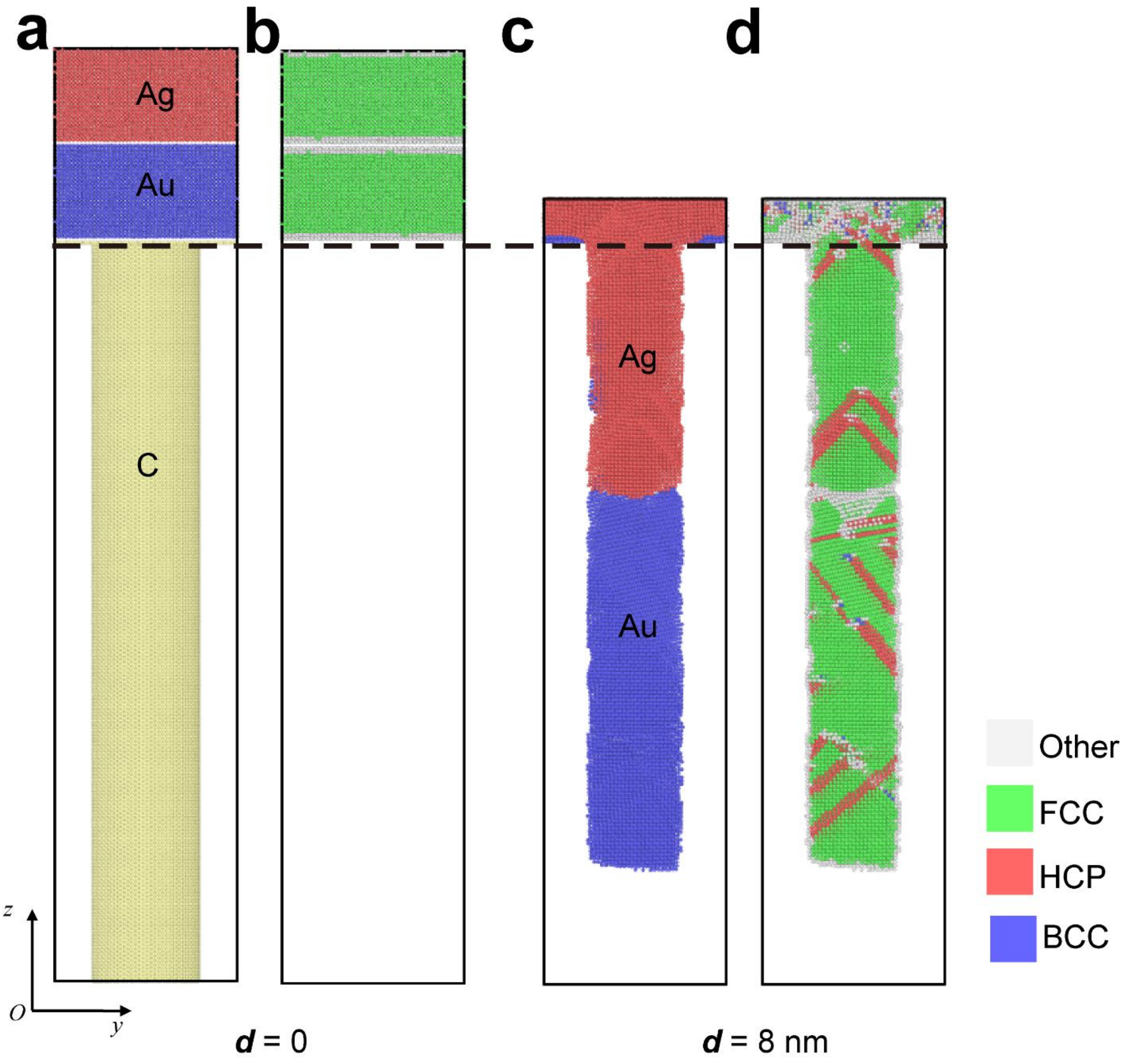

Supplementary Fig. 28 | Atomistic simulations of nanoimprinting on the Ag-Au stacked substrate under cyclic loading. a-b, Model setup and the structure type illustrations. **c-d,** Sectional views of the metal morphology and the structure type at indenter displacement of d = 8 nm. Only Van der Walls interactions was imposed between Ag and Au, consistent with the experimental observations. The heterojunction between the nanoimprinting created Ag nanowire and Au nanowire was flat and clear, which is similar to the experimental results in Supplementary Fig. 18.

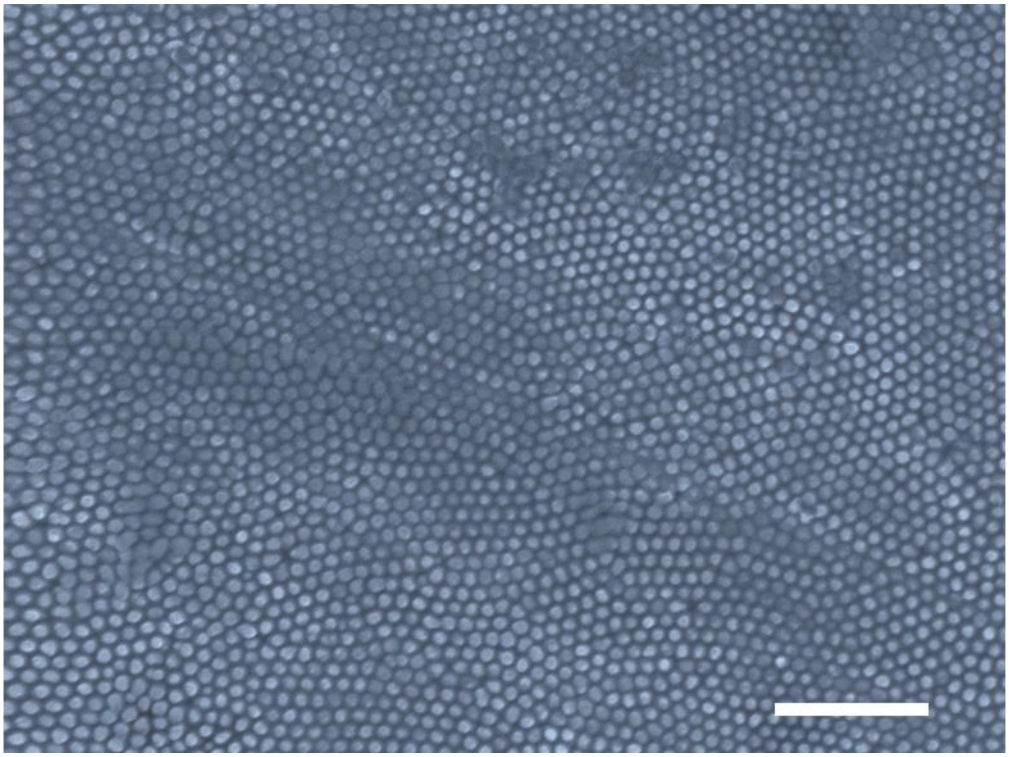

Supplementary Fig. 29 | SEM image of the pure Ag nanorod substrate. Scale bar, 1 μm.

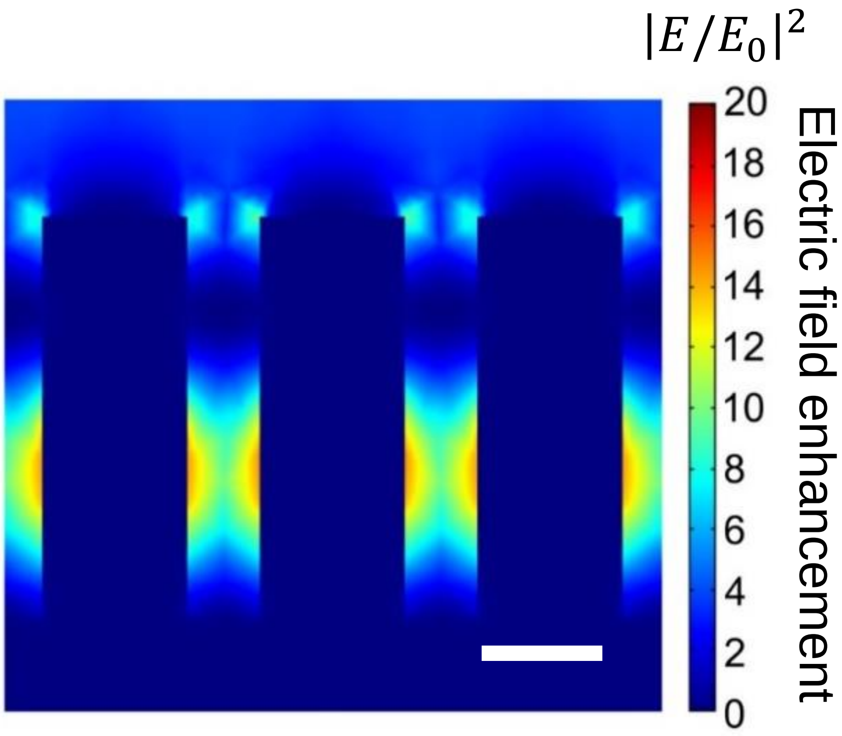

Supplementary Fig. 30 | Electric field enhancement on nanorods. Calculated electric field intensity in the pure Ag nanorod substrate. Scale bar, 100 nm. Color scale shows the calculated electric field enhancement factor.

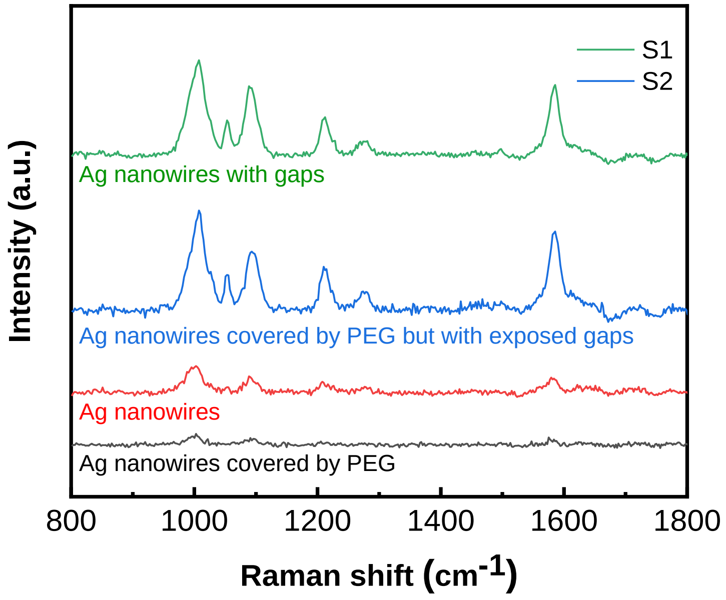

Supplementary Fig. 31 | Role of the nanogaps. SERS signal of 4-Mpy on various nanowires.

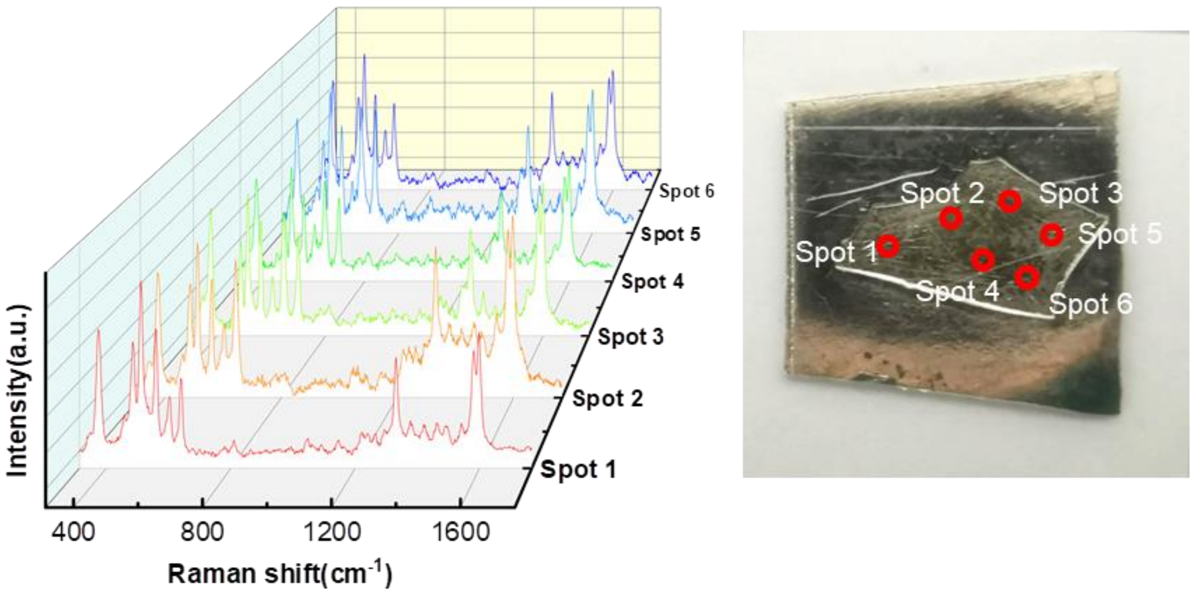

Supplementary Fig. 32 | SERS signals of six spots on one sample. Right inset: a photo of the sample.

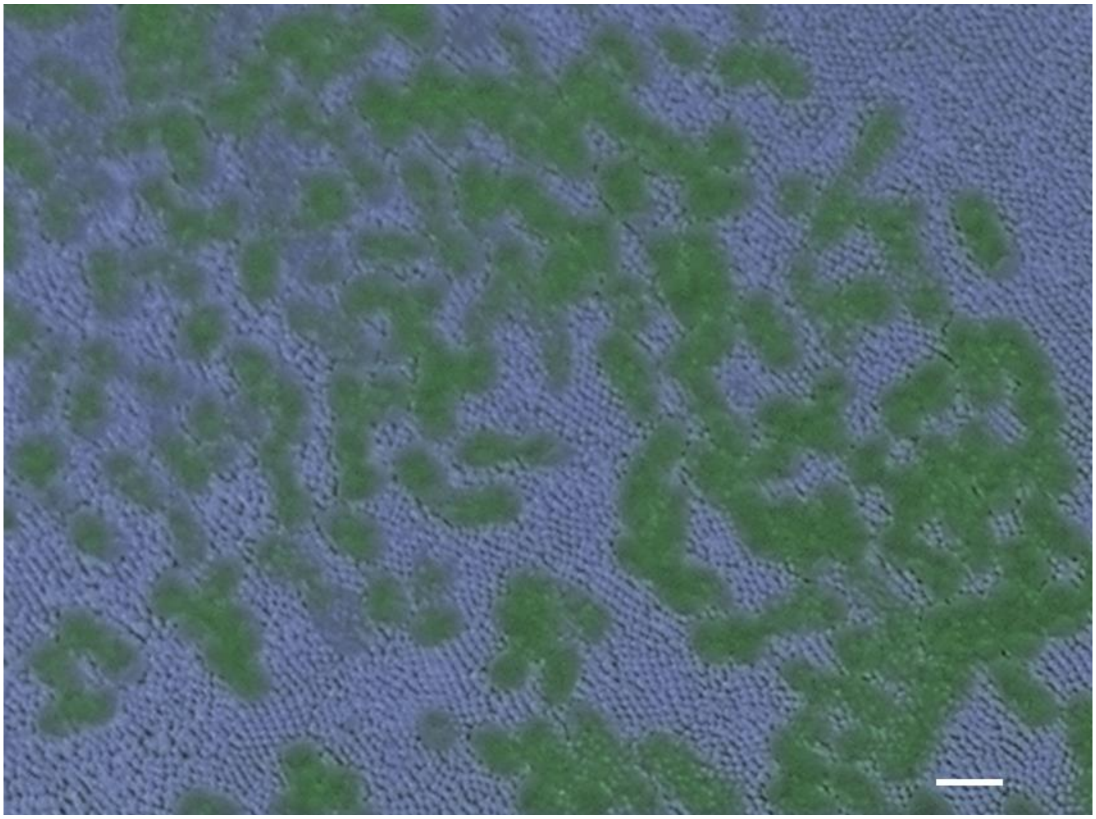

Supplementary Fig. 33 | SEM image of bacteria. *P. aeruginosa* grown on the gapped Ag nanorod substrate with 100 μg ml^{-1} erythromycin is added. Scale bar, 1 μm.

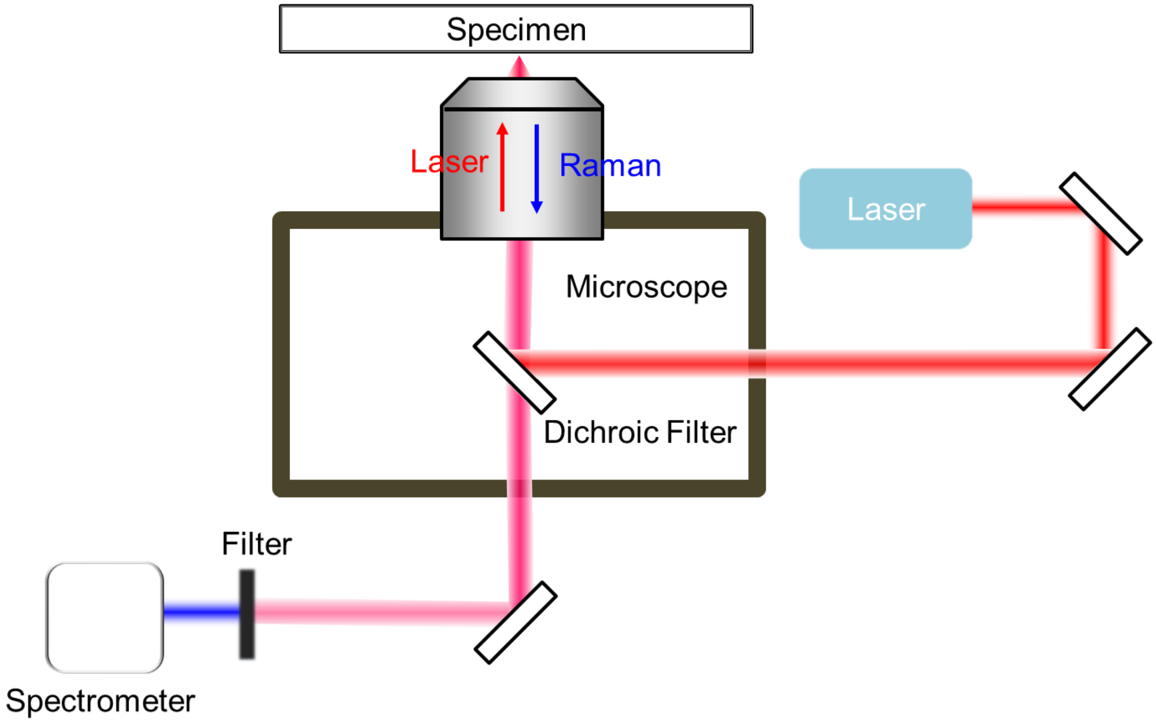

Supplementary Fig. 34 | SERS signal detection system. Schematic of Raman setup.

Supplementary Text 1. Theoretical analysis of thermal transport in the metal sample during ultrasonic imprinting

During ultrasonic imprinting in an open environment, the associated released heat mainly comes from local plastic energy dissipation at the corner. Taking Ag sample as an example, the local plastic energy induced heat transport in the NW-axial direction can be analyzed.

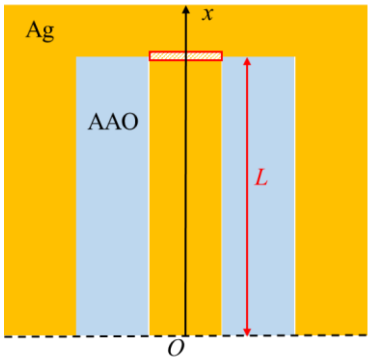

Schematic of model set up

In the metal sample, thermal transport along the axial direction can be simplified as

$$\frac{\partial u}{\partial t} = \alpha \frac{\partial^2 u}{\partial x^2}, \alpha = \frac{k}{\rho C_\mathrm{P}} \tag{1}$$

where u, t and x are the temperature, time and coordinate, respectively. Parameters α, k, ρ, C_P are used to denote the thermal diffusivity, thermal conductivity, density, and specific heat capacity, respectively. Suppose that after each cycle, all the dislocation energy W_d at the corner ($x = L$) can be harvested to contribute to the local temperature rise Δu, $W_\mathrm{d}V=\rho VC_\mathrm{P}\Delta u$. We can estimate the local temperature rise at the corner as

$$\Delta u = \frac{W_\mathrm{d}}{\rho C_\mathrm{P}} \tag{2}$$

where the dislocation energy W_d is the sum of the energy by all dislocations $W_d = \rho_d w_d$. Here, ρ_d is the number of dislocations per unit volume and w_d is the energy of a single dislocation, which includes the elastic strain energy and dislocation core energy.

$$w_{\mathrm{d}} = w_{\mathrm{el}} + w_{\mathrm{core}} = \frac{Gb^2 l}{4\pi K}\left(\ln\frac{R}{r_0} + Z\right) \tag{3}$$

where G is the shear modulus of the metal sample, b and l are the Burgers vector and the length of the dislocation respectively; $K = 1$ for screw and $K = 1-\nu$ for edge dislocations. The two items in the bracket correspond to contributions from the long-range elastic field [$\sim\ln(R/r_0) \approx 5\text{-}17$] and the dislocation core ($\sim Z \approx 1\text{-}3$), respectively. To estimate the maximum-possible temperature rise, we adopted the upper bound values of all parameters $\rho_d = 10^{16}$ m^{-2}, $w_d = 6$ eV with the Burgers vector $\mathbf{b} = 3\times10^{-10}$ m, and obtained an estimate of the maximum temperature rise $\Delta u \approx 15$ K. Then we set the boundary conditions of Eq. (1) as

$$\begin{aligned} &u(x,0) = \Delta u \sin\left(\frac{\pi}{2L}x\right) + R_0 \\ &u(0,t) \equiv R_0, \frac{\partial u}{\partial x}(L,t) = 0 \end{aligned} \tag{4}$$

where R_0 is the environmental temperature (room temperature 300 K). For an Ag NW with the maximum length of ~0.045 mm, the numerical solutions below showed that the local heat generated in one cycle is transported away quickly and has negligible effect on the next cycle.

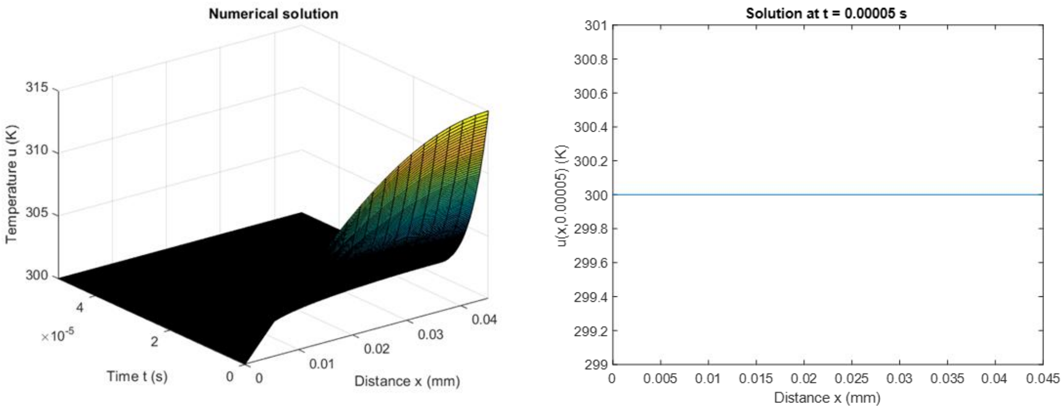

Numerical solution to thermal conduction in the NW-axial direction

Our theoretical analysis is consistent with the result from a similar experiment that has monitored the temperature of aluminum sample by an embedded thermocouple in the ultrasonic loading tests[1]. Considering the similar thermal properties of common metals as listed in Supplementary Table 4, this theoretical analysis should be applicable to a wide range of metal samples.

Supplementary Tables

AAO pore diameter (nm)	Force (N)	Amplitude (%)	Holding time (s)
300	800	20	20
80	800	18	30
50	800	15	60
20	800	10	60

Supplementary Table 1 | Parameters used for different diameter Au nanowires fabrication.

Metal	Force (N)	Amplitude (%)	Holding time (s)
**Au nanowires on silicon wafer**	300	10	20
**Sn**	600	10	20
**Al**	1000	15	30
**Bi**	800	10	30
**Ni**	1200	25	30
**Cu**	1200	25	60

Supplementary Table 2 | Parameters used for different metal nanowires fabrication.

Interaction	σ (Angstrom)	ε (eV)
**Ag-C**	3.1177	2.7e–3
**Au-C**	3.1823	2.8e–3
**Ag-Au**	2.8691	1.6e–3
**C-C**	3.4309	4.6e–3

Supplementary Table 3 | The Lennard-Jones (LJ) parameters used in MD simulations. The LJ parameters of single materials are from Ref. 2. The interactions are obtained by Lorentz-Berthelot rules.

Material	Thermal diffusivity (mm^2 s^{-1})	Thermal conductivity (W m^{-1} K^{-1})	Melting temperature (^oC)
**Ag**	166	420	961
**Au**	127	318	1063
**Cu**	111	390	1083
**Al**	97	220	660
**Plastics**	~0.1	~0.1	~200

Supplementary Table 4. Summary of the thermal properties of common metal materials and plastic materials[3,4].

Supplementary References

1. Daud, Y., Lucas, M. & Huang, Z. Modelling the effects of superimposed ultrasonic vibrations on tension and compression tests of aluminium. *J. Mater. Process. Technol.* **186**, 179-190 (2007).
2. Rappé, A. K., Casewit, C. J., Colwell, K., Goddard III, W. A. & Skiff, W. M. UFF, a full periodic table force field for molecular mechanics and molecular dynamics simulations. *J. Am. Chem. Soc.* **114**, 10024-10035 (1992).
3. Sears, F. W., Zemansky, M. W. & Young, H. D. (Wesley Publishing Company, Reading, Massachusetts, 1987).
4. Klein, R. Laser welding of plastics: materials, processes and industrial applications. (John Wiley & Sons, 2012).
