# Supplementary material for: Rapid fabrication of complex nanostructures using room-temperature ultrasonic nanoimprinting
Source: Nat Commun. 2021 May 25;12:3146. doi: 10.1038/s41467-021-23427-y (PMC8149427; doi:10.1038/s41467-021-23427-y)
Supplement: Supplementary file 3 — Description of Additional Supplementary Files [file 41467_2021_23427_MOESM3_ESM.pdf]

Description of Additional Supplementary Files

File Name: Supplementary Movie 1
Description: Video of ultrasonic nanoimprinting process. The video records metal nanowires fabrications process.

File Name: Supplementary Movie 2
Description: Atomistic simulation of nanoimprinting on Ag under direct loading (δ = 1). The video records sectional views of the deformed Ag substrate colored by the atomic local lattice symmetry. Color green, red and blue indicate FCC, HCP, and BCC types, as declared in main Fig. 3b. As the imprinting displacement increases, dislocations continuously nucleate, glide, accumulate, pile-up and interact.

File Name: Supplementary Movie 3
Description: Atomistic simulation of nanoimprinting on Ag under cyclic loading (δ = 0.14). The video records sectional views of the deformed Ag substrate colored by the atomic local lattice symmetry. Color green, red and blue indicate FCC, HCP, and BCC types, as declared in main Fig. 3b. Dislocation generation and recovery at the corner alternates during loading and retreating cycles.

File Name: Supplementary Movie 4
Description: Atomistic simulation of nanoimprinting on Ag under cyclic loading (δ = 0.05). The video shows sectional views of the deformed Ag substrate colored by the atomic local lattice symmetry. Color green, red and blue indicate FCC, HCP, and BCC types, as declared in main Fig. 3b. Dislocation generation and recovery at the corner alternates during loading and retreating cycles.

File Name: Supplementary Movie 5
Description: Temperature measurement during ultrasonic nanoimprinting. The video records the temperature change of Ag metal foil during ultrasonic nanoimprinting. The loading force is 1,200 N. The amplitude is 25% in this process. A very thin and sensitive thermocouple was stuck on the metal foil in this process.
